# Predicting Ion Diffusion from the Shape of Potential Energy Landscapes

**DOI:** 10.1021/acs.jctc.3c01005

**Published:** 2023-12-19

**Authors:** Hannes Gustafsson, Melania Kozdra, Berend Smit, Senja Barthel, Amber Mace

**Affiliations:** † Department of Chemistry—Ångström, Uppsala University, Uppsala SE-751 21, Sweden; ‡ Institut des Sciences et Ingénierie Chimiques, Valais, Ecole Polytechnique Fédérale de Lausanne (EPFL), Rue de l’Industrie 17, Sion CH-1951, Switzerland; § Department of Mathematics, Vrije Universiteit, Amsterdam 1081 HV, Netherlands

## Abstract

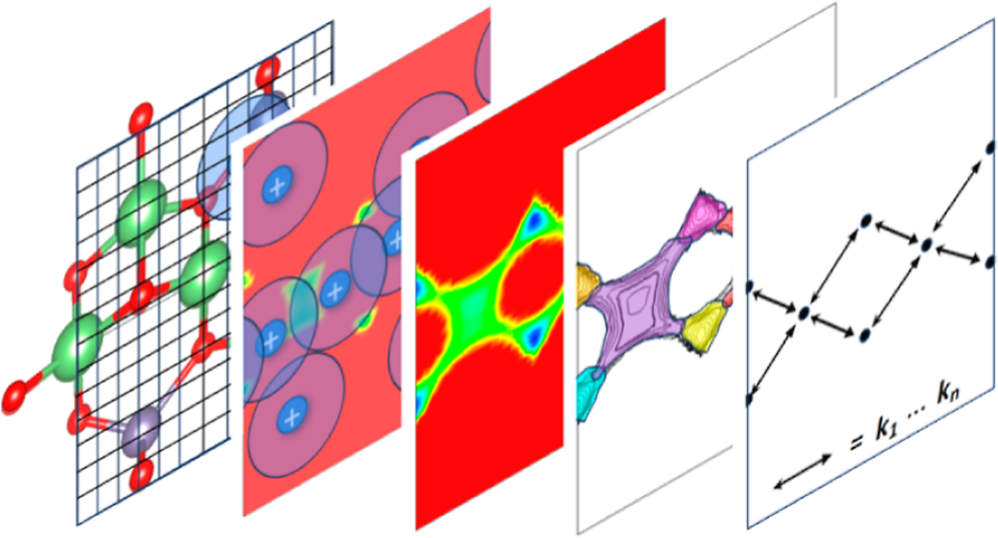

We present an efficient method to compute diffusion coefficients of multiparticle systems with strong interactions directly from the geometry and topology of the potential energy field of the migrating particles. The approach is tested on Li-ion diffusion in crystalline inorganic solids, predicting Li-ion diffusion coefficients within 1 order of magnitude of molecular dynamics simulations at the same level of theory while being several orders of magnitude faster. The speed and transferability of our workflow make it well-suited for extensive and efficient screening studies of crystalline solid-state ion conductor candidates and promise to serve as a platform for diffusion prediction even up to the density functional level of theory.

## Introduction

Mass transport is a fundamental component in a wide range of chemical and physical phenomena, e.g., chemical reactions in condensed phases, the absorption of molecules in porous materials, and ion conduction in electrolytes. To understand these phenomena, theoretical insight into the underlying microscopic mechanisms is required. This can often be achieved by interpreting the results of computational modeling at an atomic level. In particular, computational studies allow for exploring the vast chemical and structural space to an extent not achievable by other means to both deduce structure–property relationships and screen for promising materials for a given target application. For screening studies, it is useful to have a low-cost computational method that can reliably discard materials that are not of interest, leaving a small number of promising materials to which more costly computational or experimental efforts can be dedicated.

Transport properties, such as diffusion, are usually computationally modeled using molecular dynamics (MD). This well-established method suffers from a high computational cost for diffusion that is determined by sequences of separate discrete “rare event” moves of the diffusive particles. To be able to study large numbers of materials with this type of diffusion, for both qualitative understanding as well as for finding promising materials, more efficient and sufficiently accurate strategies for the computational prediction of diffusivity are needed.

Material classes that require an efficient methodology to compute diffusion coefficients include crystalline solid-state materials that are relevant to energy applications. Several of their diffusion properties rely on interstitial and vacancy-mediated diffusion, e.g., gas diffusion in nanoporous materials and ion diffusion in solid-state electrolytes (SSEs) for Li-ion and Na-ion batteries. In the crystalline solid-state, where the energy landscape can be considered mostly fixed due to the rigid arrangement of atoms, computational simulations of diffusion can exploit the dependence of the diffusion of a particle on the potential energy surface (PES) that the particle experiences as it moves. This idea was utilized in previous work by some of the authors in the development of the Tunnel and Transition State (TuTraSt) algorithm,^1^ in which a general topological and geometrical analysis of the PES felt by a migrating particle is performed. The method was tested on CH_4_ diffusion in zeolites and was shown to accurately predict diffusion coefficients in 96% of the tested structures at a fraction of the computational cost of MD.^1^ Using this topology- and geometry-based approach to simulate ion diffusion in SSEs requires the introduction of an additional routine to the workflow used in the methane study to account for loading effects of interacting particles.

Li-ion batteries are widely used as energy storage solutions. However, their importance for energy-transition applications such as in electric vehicles demands further improvements,^2^ in particular addressing the safety issue arising from the flammability of the liquid organic electrolytes that are currently used in batteries. SSEs have the potential to be safer, as well as more time stable compared to liquid electrolytes. Any plausible new electrolyte material needs to show a high ion conductivity, allowing Li ions to diffuse with low resistance from cathode to anode and back upon charging and discharging. The typically slow ion diffusion in the solid state is a prohibitive factor when considering new candidates for SSEs, which must achieve similar specific ion conductivity values as liquid electrolytes that currently reach >0.01 S/cm at room temperature.

Only a few highly conductive crystalline materials have been revealed to date, and the mechanisms promoting high ion conductivity in these so-called superionic conductors are not yet fully understood. Candidates have been found in a diverse range of structural families, including LISICONs (Li superionic conductors), NASICONs (Na superionic conductors), perovskites, antiperovskites, garnets, argyrodites, and sulfides.^2–6^

One promising strategy to optimize the conductivity of experimentally identified candidates is through compositional tuning.^7,8^ In addition, from a fundamental point of view, systematically studying structural derivatives and introduced variations in existing structures at a large scale would further help to uncover the mechanisms, general principles, and structure–property relationships for Li-ion conductivity in inorganic solids. At the same time, the set of potential structures applicable to SSEs is huge, and although an exhaustive and systematic study of existing and hypothetical materials would greatly aid the discovery of more highly conductive candidates, scientific and practical challenges in tackling such a task remain.

Recent efforts to screen materials for ion conductive crystalline inorganic materials through computational modeling have been made using different approaches.^9–13^ However, hitherto, the methods used typically fall short either in required accuracy, speed, or in the possibility to automate, e.g., due to limitations in applicability or transferability between structural families.

An approach based on topological and geometric analysis of the PES, such as the TuTraSt algorithm, has the advantage that it is unlimited to structural characteristics of the material, including the type of the migrating ions, e.g., any cation (Li^+^, Na^+^, Mg^2+^, etc.). This allows screening of a wide range of structures across many material families (perovskites, garnets, etc.). Moreover, the accuracy and speed of the approach depend mainly on the level of accuracy the potential energy is computed at, which can be done using, e.g., force fields, density functional-based tight-binding, or density functional theory (DFT).

TuTraSt’s direct estimation of diffusion coefficients from PESs circumvents computationally expensive MD simulations for slow diffusing particles. Also, it allows for a quick rejection of nondiffusive structures, which often form the majority in a screening study. We consider a material to be nondiffusive if its diffusion coefficients are below a lower limit. Here, this can be chosen as the lowest coefficients that can be obtained by a computational method or as the minimal diffusivity needed for a given application. The characteristics of Li-ion diffusion in crystalline inorganic SSEs make it a very suitable application of the TuTraSt approach. However, in contrast to the previously studied case of gas diffusion in zeolites, where the interactions between the migrating gas molecules are weak and mainly consist of local collisional and channel-blocking interactions, the Coulombic ion–ion interactions are not negligible. These introduce potentially strong loading effects that significantly affect the diffusion. It is therefore vital to include these interactions to adequately model the transport of ions, in contrast to the case of weakly interacting gas molecules, where the single-particle energy provides an accurate description of the free energy landscape even at higher loading.

In this work, we develop Ionic TuTraSt, an adaptation of the TuTraSt algorithm,^1^ in which ion–ion interactions are taken into account as a correction to the single-particle energy. A Metropolis Monte Carlo scheme is applied to incorporate the loading effects due to the strong electrostatic interactions between the migrating ions. This leverages the advantages of the TuTraSt approach and extends the applicability to systems with strongly interacting migrating species such as diffusing ions in inorganic solids.

As a case study, we apply the introduced Ionic TuTraSt procedure to a database of Li-containing crystalline inorganic SSE candidates and validate the resulting diffusion coefficients against MD. As expected, the proposed correction significantly improves the result compared to TuTraSt with only the single-particle grid. We find that Ionic TuTraSt not only rapidly identifies the nondiffusive structures correctly but also predicts diffusion coefficients in agreement with MD within 1 order of magnitude in >98% of the cases.

These results show that the Ionic TuTraSt is a general, fast, and accurate procedure for predicting diffusive and nondiffusive crystalline inorganic ion conductors and offers a framework that opens up for predicting ion diffusion even at the density functional level of theory. Thus, our method can contribute to enabling more extensive screening studies of ion conduction in crystalline solids and efforts to identify conductive candidates for inorganic SSE materials in Li-ion or Na-ion batteries.

## Theory and Methodology

### Ion Transport in Inorganic SSEs

Li-ion transport within inorganic SSEs occurs through interstitial lattice diffusion mechanisms, where the ion migrates through jumps between vacant interstitial sites. The hopping type motion between lattice vacancies arises from the well-defined structure of the solid and the relatively high energy barriers separating the vacancies, which are typical for ion diffusion in solids and are the main culprit for the often slow transport. Consequently, the high barriers lead to a separation of the time-scales associated with the dynamics within the interstitial Li sites and the transitions between them. These transitions between Li sites are related to the macroscopic transport. In other words, site-to-site transitions become so-called “rare-event” processes relative to the within-site dynamics.^14^ To simplify, the diffusion rate depends on the availability of vacant sites and the height of the energy barriers between the sites.

Many studies have focused on structural tuning strategies aimed to affect these two parameters to improve and optimize the conductivity of specific structures. Such strategies include optimizing the ion/vacancy ratio through aliovalent substitution or introducing structural defects or by altering the topology of the conduction channels through the framework to decrease the energetic and structural bottlenecks and optimize the minimal energy pathway.^7,8^ In many cases, this has shown to be a fruitful strategy where compositional tuning can improve the conductivity up to 6 orders of magnitude within a structural family.^6^ Despite having identified these mechanisms, it is not straightforward to predict the optimal material or how to apply strategies effectively as the effects of tuning vary widely between structures and even more between structural families. Systematic and extensive studies across a wide range of structural compositions are needed as the trial-and-error design approach of today has not been sufficient to understand the universal trends and mechanisms driving the conductivity in the diverse chemical and topological space of ion-conducting materials.

### State of the Art: Modeling Ion Diffusion in Crystalline Inorganic Electrolytes

To study ion migration computationally, the state-of-the-art approach is to perform MD simulations, which is based on the stepwise solving of Newton’s equations of motion.^15^ This is an elegant and explicit way to model the dynamics, but to compute the diffusion for a specific system, sufficiently long simulations are required.

To be accurate, MD simulations necessarily must resolve the shorter time-scale dynamics of the ions within the interstitial sites. At the same time, the simulations must be long enough to capture the longer time-scale and specifically to be able to reach the diffusive regime corresponding to the site-to-site dynamics responsible for the transport. This leads to poor statistics of the intersite transitions and, hence, of the diffusion process and increases the required computational time, especially for materials with slow diffusion. Moreover, most of the simulation time is spent sampling the ion dynamics within the interstitial sites, which does not contribute to the transport.

For studying the diffusion of a limited number of structures and using inexpensive preparameterized force fields, this approach is reasonable. However, when looking at larger numbers of structures, this computational cost will quickly become impractical, particularly for systems with a slow diffusion. In addition, it is often necessary to use more advanced methods, such as DFT, to estimate the interatomic forces, making atomic-scale MD simulations for Li-ion conductors practically impossible. Simplifying DFT methods can mitigate the time-scale limitations of MD while still taking advantage of higher theory-level methods. However, even when using current developments such as the Pinball-MD model^11^ and allowing high computational costs, only the fastest diffusing materials can be identified: A screening of ∼900 Li-containing structures from crystal structure databases^16,17^ using 14 M CPU hours could obtain converged diffusion coefficients for about ∼3% of the structures.^10^ This low convergence is a direct consequence of the low diffusion coefficients. An absolute lower bound for the required CPU time is that a Li ion should diffuse through at least one unit cell. The average unit cell length in the screening study is 11 Å (in the range of 6–27 Å), and the simulation time for each structure is 100–200 ps. Hence, for diffusion coefficients smaller than 10^–5^ cm^2^/s, one needs significantly longer trajectories.

For diffusion in solids, where the transport processes can be considered rare events and process rates can be separated and approximated, kinetic Monte Carlo (kMC) can be a powerful tool to combine these separate processes and thus predict the overall diffusion for systems where this is otherwise difficult or impossible to probe with MD. The periodic nature of crystalline inorganic SSEs deems lattice-based kMC simulations to be particularly appropriate to study Li-ion diffusion and has, in the past few years, become a quite popular approach to study ion transport in SSEs. The state of the art is to rely on nudged elastic band (NEB)^18^ calculations to compute the energy barriers for the ion hopping between interstitial sites.^15^ However, NEB calculations require prior knowledge of the positions of the sites, as well as directions of the conduction channels, which often entail tedious studies of specific structures that can be transferred only within structural families, such as has been done for antiperovskites^12^ and NASICONs.

### Topology- and Geometry-Based Analysis

The TuTraSt algorithm depends on a topology- and geometry-based analysis of the potential energy landscape felt by the diffusing particle within the host structure only. This circumvents the previously discussed drawbacks inherent to MD and NEB. TuTraSt partitions the PES into energy basins and the transition states between them, from which it constructs an accurate lattice-kMC-based diffusion model by applying transition-state theory. The geometrical partitioning algorithm in TuTraSt is rigorous in finding all types of channels and transition-state surfaces by employing a sequential procedure where basins are grown carefully in a stepwise fashion by traversing the energy isolevels in increasing order. The transition states can be identified as the merging points of the basins and additionally yield information about the direction, dimensions, connectivity, and barrier heights of the channels. By integrating the regions identified as basins and transition states, the rate constants for the transitions are obtained through the Bennett–Chandler approach.^19^ Compared to NEB, the benefits of this approach are that geometry and topology are not required to be known beforehand, which makes it easily applicable to, in principle, any structure and greatly facilitates automation. Moreover, only the PES is required and no calculations of forces are needed. The free energy surface provided as input to the geometrical analysis routine is not limited to a specific model or method but can be obtained at any desired level of theory. This thus provides a scheme where the diffusion, in principle, can be predicted at the DFT level in systems that would not be feasible with ab initio MD due to time-scale limitations.

In cases where diffusivity is low and hence the activation barrier for diffusion is high, diffusion channels arise only at a higher energy. This information is captured in the energy landscape. If the energy value at which channels are formed is not thermally reachable, a topological and geometrical analysis of the energy landscape can immediately exclude nondiffusive materials. This is a significant benefit over MD, where the question of whether the diffusive regime has been reached needs to be evaluated based on the trajectory and cannot be determined prior to a simulation. Potentially long simulations thus need to be conducted even for poorly diffusive candidates.

The method has been validated for experimental accuracy for noncharged particles diffusing at low loadings in porous materials. However, while predicting the diffusion in such systems can be done from a single-particle grid, simulating ionic transport in SSEs requires significant development of TuTraSt to include the effects of multiparticle interactions. This is described in the following sections.

### Ion–Ion Interaction Correction

The single-particle approximation of the PES can appropriately describe charge-neutral methane molecules diffusing within a nanoporous material. However, when studying SSEs, a single-particle grid is not sufficient since the strong ion–ion electrostatics at high concentrations of positively charged ions must be taken into account. Conceptually, from the perspective of the migrating ions, the potential energy is partitioned into two components: The first component is a single-particle component, *E*_SP_(***r***), which is the potential energy of a single ion at position ***r*** within the framework structure. In this calculation, the framework structure is taken as the material, with all migrating ions removed. In other words, the single-particle component arises from the interactions between one migrating particle and all immobile atoms. The second component of the potential energy is an interaction component, *E*_ion–ion_, which is due to interactions between migrating ions. This is taken as the pairwise interactions between the migrating particles, consisting of their electrostatic and van der Waals interactions.

Note that since we treat the framework as fixed, the contribution of interactions between framework atoms is a constant shift of the PES, which does not affect the barriers associated with moving on the surface. Hence, it does not affect the migration dynamics in this model and thus can be neglected. With these assumptions, we obtain the multiparticle potential energy
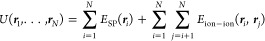
1

**Figure 1:**
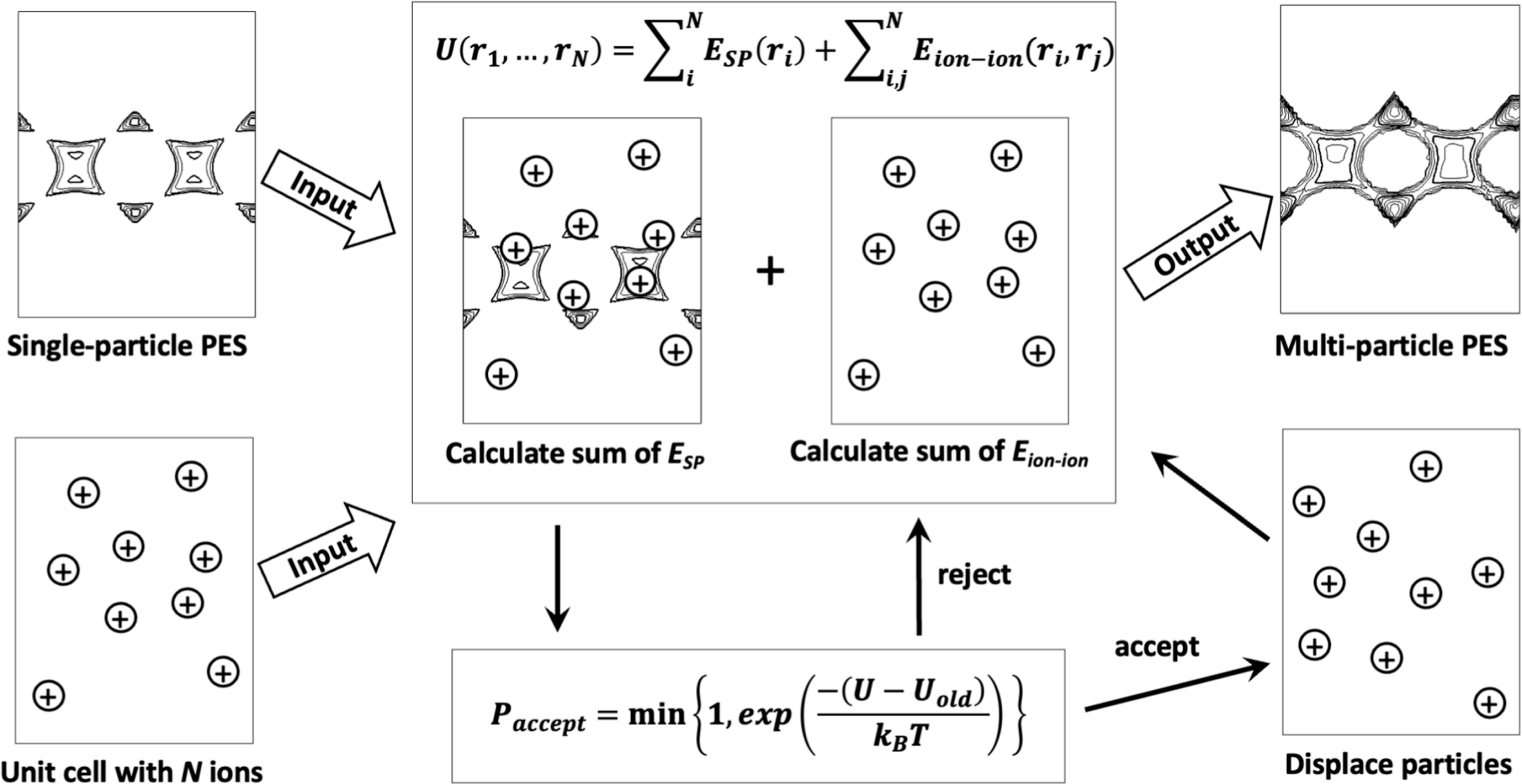
Schematic outline of the MMC procedure, carried out to compute the multiparticle PES. The input to the MMC scheme is the single-particle PES grid (top left) and the simulation cell containing only the stoichiometric number of ions (*N*) of the structure (bottom left). The configurational energy sampled at each MMC step is a sum of the tabulated single-particle energies (*E*_SP_) of all ions and the sum of all ion–ion pair interactions. During the MMC simulations, only the ion–ion interaction energies are thus computed explicitly. These calculations are performed with a constant number of particles, volume, and temperature.

To sample *U*, we introduce a Metropolis Monte Carlo (MMC)-based approach that explicitly computes the ion–ion interactions and displacements in the canonical ensemble (*NVT*). The MMC routine is schematically presented in Figure 1 and is carried out according to the following steps:

1. Simulation setup: The number of ions corresponding to the stoichiometric content of the respective structure is loaded into the simulation cell, which is an empty supercell of a structure of size big enough to respect the minimal image convention (framework atoms are not modeled explicitly). The single-particle PES, *E*_SP_, is also read as an input.
2. Sampling of the multiparticle PES: At each MMC step, the ions are randomly displaced and the potential energy for the configuration is calculated. The total configurational potential energy *U*(***r***_1_, ..., ***r***_N_) is computed as the sum of the single-particle energies (*E*_SP_) and the pairwise interactions between the ions (*E*_ion–ion_), according to (eq 1). The interaction *E*_SP_(***r***) felt between an ion and the framework at a given point ***r*** is included by reading the position-dependent value from the precomputed grid *E*_SP_. The interactions *E*_ion–ion_ are computed explicitly in each step, using Ewald summation for the electrostatic interactions.

Once the energy has been computed, the displacement is accepted or rejected according to the MMC acceptance criteria following the Boltzmann distribution law. The canonical ensemble is withheld by keeping the number of ions (*N*), simulation cell coordinates and volume (*V*), and target temperature (*T*) constant throughout the simulation. The resulting trajectories are stored
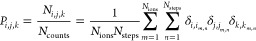
2and then converted to the multiparticle potential energy grid through (eq 3).
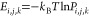
3here *E*_*i*,*j*,*k*_ and *P*_*i*,*j*,*k*_ are the multiparticle potential energy and probability density, respectively, of ions at the grid point with coordinates ***r*** = (*i*, *j*, *k*), *N*_*i*,*j*,*k*_ is the number of counts an ion has occupied at the position ***r***_*i*,*j*,*k*_ in the trajectory, *N*_ions_ is the number of ions in the simulation, *N*_steps_ is the total number of frames in the trajectory, and *N*_counts_ = *N*_ions_ × *N*_steps_. The *m*-th ion of the *n*-th frame has coordinates (*i*_*m*,*n*_, *j*_*m*,*n*_, *k*_*m*,*n*_), and δ denotes the Kronecker delta.

3. Generation of the multiparticle PES grid. The trajectory output from the previous step is converted to a new potential energy landscape by first calculating the normalized ion probability density according to (eq 2)

We implemented this MMC setup in the RASPA^20^ computational framework, where we adapted RASPA’s MakeGrid-module to import external grid data.

### Ionic TuTraSt Workflow

Applying TuTraSt to the corrected PES allows the computation of diffusion coefficients. We refer to the entire computational workflow as Ionic TuTraSt. The incorporation of the MMC procedure provides Ionic TuTraSt with a strategy to take into account interactions between the mobile ions with the aim of enabling more accurate diffusion prediction at higher mobile particle concentrations.

**Figure 2:**
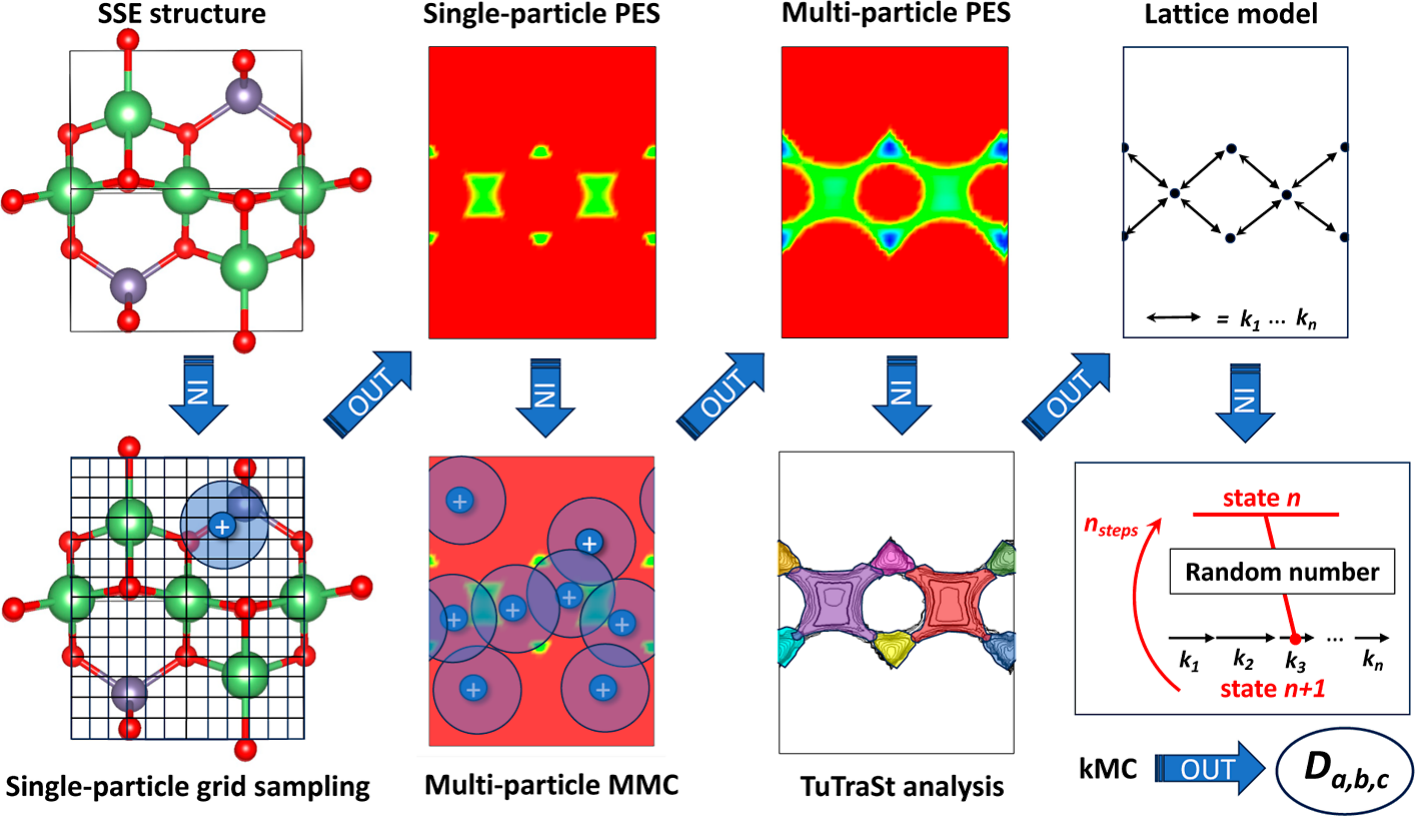
Schematic outline showing the subsequent steps in the Ionic-TuTraSt workflow. The workflow consists of four modeling routines (bottom row) that generate a single-particle PES and a loading-corrected multiparticle PES, and decompose the latter to obtain a lattice model on which a kMC simulation is performed. The input and output data formats are shown in the top row. The initial input data are the SSE structure, and the final outputs are the ion diffusion coefficients *D*_*a*,*b*,*c*_ in the directions of the cell vectors *a*, *b*, and *c*.

Its steps are described below and are schematically shown in Figure 2.

1. Constructing the single-particle PES with grid sampling. A description of the crystalline structure is provided as input to the workflow, which serves as the basis to determine the framework structure, the stoichiometric number of mobile ions, and the supercell. The single-particle PES of the migrating species, *E*_SP_, is sampled by placing a single migrating ion inside the empty framework and evaluating the energy of the resulting configuration for each grid point of a regular grid of the unit cell.
2. Constructing the multiparticle PES with MMC: The single-particle PES grid and the stoichiometric number of migrating ions are provided as input to perform an MMC simulation that samples configurations of a stoichiometric number of interacting mobile ions in the single-particle energy landscape. The MMC procedure is described in detail in the previous section. The mobile ion probability distribution and, in turn, the effective multiparticle PES, *E*_MP_, of the migrating species at stoichiometric concentration is calculated from the MMC trajectories.
3. Constructing the lattice model with TuTraSt analysis: The TuTraSt algorithm is applied to the multiparticle PES, which, through geometrical and topological analysis, finds basins, transition states, and their connectivity in the multiparticle PES. This information is used to construct a lattice model with lattice site coordinates, possible transitions between lattice sites, and corresponding transition rates.
4. Computing diffusion coefficients with kMC: The lattice model is used as input for performing kMC simulations. The diffusion coefficients of the migrating ions are computed from the resulting kMC trajectories.

## Case Study: Lithium-Ion Diffusion in Crystalline Inorganic Electrolytes

To test the performance of Ionic TuTraSt, the algorithm is applied to two sets of Li-containing inorganic crystalline materials and validated against equivalent MD simulations. The aim is to compare the methods as a means to simulate the time evolution of the systems for the purpose of computing diffusion coefficients. To allow for a valid comparison of the computational methods in this manner, it is crucial that the interaction models are identical in both methods. The first, more extensive validation set consists of 83 structures, using identical classical force field parameters for all calculations. While the force field parameter applied here, the universal force field^21^ (UFF), is not known for its experimental accuracy in the prediction of diffusion coefficients, it is parametrized for all elements. This is sufficient as the focus at this stage is not on the experimental accuracy.

Performing this comparison assumes that the relative performance of the methods can be transferred between different interaction models. Therefore, to test this, the second validation set consists of 9 highly diffusive structures for which Kahle et al.^10^ computed diffusion coefficients with the Pinball-MD^11^ methodology, a DFT-based approach. The detailed procedure for these case studies is described below.

### Validation Set 1: Force Field Grids

#### Structure Selection

From the Materials Cloud database,^22^ we limit the selection to structures containing three or four elements, including Li. Partial charges are computed for the first 100 structures by imposing the even electron criterion to avoid more time-consuming spin-polarized DFT calculations. The final validation set consists of 83 structures.

#### Force Field Parameters

To obtain comparable results, interactions are described within the same model throughout, i.e., in all MD simulations, sampling of single-particle grids, and MMC simulations: We use a classical force field comprised of Lennard–Jones (LJ) potentials with parameters from the UFF^21^, together with Coulomb potentials computed through the standard Ewald summation approach with REPEAT^23^ partial charges. Details of the single-point DFT calculations for determining the REPEAT charges can be found in the Supporting Information. The Lorentz–Berthelot mixing rules are used to account for heteroatomic LJ interactions. A 12.5 Å cutoff is used for all LJ interactions. The supercells used in the Ewald summation are constructed based on the same cutoff to fulfill the minimum image convention. The same supercells are used in the MD simulations and the single-particle grid sampling. We use the *LAMMPS Interface* program^24^ to assign the force field parameters and to build supercells. All diffusion coefficients are computed in the direction of the cell vectors.

#### MD Computational Details

For the 83 structures in validation set 1, we run classical MD simulations using the LAMMPS package.^25^ All simulations are performed within a rigid framework, i.e., atoms other than Li are kept fixed and periodic boundary conditions are applied in every dimension. The MD simulations are run with a 1 fs time step to obtain 250 ns trajectories in the *NVT* ensemble. The temperature is controlled by a Nose–Hoover thermostat at 1000 K.

The resulting Li trajectories with coordinates printed every 1 ps are used to calculate the mean-square displacement (MSD) computed along the unit cell vectors. The MD diffusion coefficients (*D*_MD_) are obtained from the slopes of the ensemble-averaged MSDs: The slope of the MSD curves is calculated from the region between the points at which the root-mean-square displacement (RMSD) surpasses one and two lengths of the respective cell vector (***a***, ***b***, ***c***). The criterion that the RMSD should go beyond the respective cell parameter is considered a lower bound and necessary condition for reliably observing diffusion in the simulation, and *D*_MD_ is set to 0 in cases where this is not met. Effectively, we interpret this as a sign that Li does not diffuse within the time-scale of the MD simulation. Additionally, we verify that the slope of the MSD as a function of *t* on the log–log scale equals 1 to ensure that the diffusive regime indeed has been reached.

We propose that the lower limit of the diffusion coefficient corresponds to a distance traveled of at least one unit cell. In that very optimistic assumption, the minimal diffusion coefficient that can be computed, from the given 250 ns trajectory of Li ions in crystals that have unit cells between 3.3 and 14.7Å, is in the range of 1.6 × 10^–8^–3.2 × 10^–7^ cm^2^/s.

#### Single-Particle PES Grid Sampling

For each of the 83 structures, the three-dimensional single-particle potential energy grids (*E*_SP_) are computed on regular rectilinear coordinate grids in fractional coordinates, with voxel size 0.2 × 0.2 × 0.2 Å^3^. The energy of each grid point is computed as the energy of the configuration that is obtained by placing a single Li atom at the position of the center of the corresponding voxel in the empty framework. The single-particle potential energy sampling is implemented in Python and utilizes the Atomistic Simulation Environment (ASE) Python package.^26^

Before starting the MMC simulation, we precompute the single-particle grids in the cube format. The unit cube grid coordinates are then mapped to a cubic simulation cell in the cell vector basis. In this format, the grids are then read by a modified version of the RASPA MakeGrid-module and converted into the RASPA grid format that can be used by RASPA for the MMC simulation.

#### Multiparticle PES Grid Sampling

MMC simulations in the *NVT* ensemble are performed as described in the previous section. In every simulation, the MMC moves are performed on the stoichiometric number of Li ions within a supercell box, corresponding to an empty supercell of the given structure, with a size determined to fulfill the minimum image convention. The energy is calculated as the sum of the contributions from the Li particles due to the single-particle grid *E*_SP_, which is read from the input, and the interactions between the Li particles, which are calculated in the simulation.

Periodic boundary conditions are imposed in each direction. The simulations consist of an equilibration phase of 100,000 MMC cycles, followed by 1,000,000 production MMC cycles. Here, the term “cycle” is used as in RASPA: a cycle consists of *n* trial moves, either accepted or rejected, where *n* = max(20, number of particles moved in the simulation). The temperature in the MMC runs is set to 1000 K. From the MMC trajectories, the Li probability density distribution is calculated using eq 2, from which the corrected multiparticle energy grid *E*_MP_ is obtained using eq 3.

#### TuTraSt Analysis to Obtain PES-Based Diffusion Coefficients

The TuTraSt algorithm is applied to both the *E*_SP_ and *E*_MP_ grids, producing the single-particle diffusion coefficients *D*_SP_ and the multiparticle diffusion coefficients *D*_MP_, respectively. An energy step of *E*_step_ = 1.0 kJ/mol and an energy cutoff of *E*_cutoff_ = 100.0 kJ/mol are used in the TuTraSt analyses.^1^ Practically, this cutoff energy means that diffusion processes associated with barriers over 100.0 kJ/mol are not considered, as they will have a negligible impact on the diffusion at the temperatures considered here. If no diffusion channels are found below this energy, then the diffusion coefficient is set to 0. The energy step dictates the resolution of levels in the TuTraSt analysis and is related to the minimum barriers that can be resolved.^1^ For every energy grid, five kMC simulations of 250,000 steps each are run for temperatures 300, 500, 700, and 1000 K. The diffusion coefficients in the direction of the cell vectors and their standard deviations are computed as the average over the simulations at the corresponding temperature. This results in 249 so-called directional diffusion coefficients.

#### Validation of Temperature Dependence

For the structures that are predicted to have fast Li diffusion (*D*_MP_ > 10^–6^ cm^2^/s) at 300 K in TuTraSt analysis, we run additional MD simulations at 300, 500, and 700 K, using the same settings as in the previous MD simulation except for setting the Nose–Hoover thermostat to the corresponding temperatures. From the diffusion coefficients at different temperatures, we plot Arrhenius plots based on the TuTraSt and MD results, respectively, to compare their temperature dependence.

#### Validation Set 2: Pinball Grids

From the recent work by Kahle et al., we identify nine of the fastest diffusing structures having sufficiently converged trajectories to provide a solid base for validation. For these nine structures, the Pinball-MD trajectories and 0.2 × 0.2 × 0.2 Å^3^ single-particle PES grids computed with the Pinball potential are both provided by the authors.

The single-particle Pinball PES grids are used as input for computing multiparticle diffusion coefficients using an Ionic TuTraSt procedure identical to what is carried out for validation set 1 at *T* = 1000 K. However, given that the MMC loading correction module is carried out using classical force fields that require assigning partial charges, whereas the Pinball calculations are based on DFT potential energies, the partial charges and interaction parameters for the Li–Li pair potentials used in the MMC loading correction must be fitted against the DFT-based calculation to ensure consistency. As the short-range electrostatic repulsion between the Li ions is expected to be dominant over the dispersion energies, UFF LJ parameters are simply applied while a range of partial charges are tested; *q* = 0.25*e*, 0.5*e*, 0.75*e*, and 1*e* as well as a set of charges determined by the REPEAT method computed with the same procedure as for the structures in validation set 1. Of these charge sets, *q* = 0.5*e* shows the best agreement with the Pinball-MD data, as presented in Figure S2.

## Results and Discussion

The Ionic TuTraSt workflow provides a significant correction compared with single-particle TuTraSt for both validation sets. For the respective sets, the prediction accuracy is brought up to 98 and 100% of the diffusion coefficients when adding the multiparticle MMC scheme to the workflow. Although the MMC scheme adds significant computational cost to the Ionic TuTraSt procedure, the computational load is, on average, ∼25 times faster than that for MD when using identical force field functions.

### Validation Set 1: Force Field Grids

We compare the diffusion coefficients computed with TuTraSt analysis using the single (*D*_SP_) and loaded corrected multiparticle (*D*_MP_) grids, respectively, with those computed with the full-resolution MD (*D*_MD_).

The MD simulation at 1000 K identifies 15 out of the 83 structures (20%) to be diffusive with diffusion coefficients ranging from 10^–4^ to 10^–7^ cm^2^/s where the lower range corresponds approximately to the time-scale limit of the MD simulations. Most of these structures show diffusion in all three directions (12 structures), three structures show diffusion in two directions, and none of the structures in the validation set show one-directional diffusion according to MD. This results in a total of 42 directional diffusion coefficients, where the remaining 207 structural directions measured showed to be nondiffusive or not able to reach the diffusive regime within the time-scale of the MD simulation.

When comparing diffusion coefficients predicted by TuTraSt (single-particle as well as Ionic TuTraSt) to the ones obtained by MD, the prediction is considered to be correct if the TuTraSt value is within 1 order of magnitude of the corresponding MD value. In particular, a structure is considered nondiffusive by TuTraSt analysis if it predicts a diffusion coefficient of <10^–6^ cm^2^/s, since this is 1 order of magnitude above the lower limit of <10^–7^ cm^2^/*s* for computing diffusion coefficients from the MD simulations for most structures studied. If a structure is considered as nondiffusive, its diffusion coefficient is set to zero.

In correspondence with the MD data, the majority of the structures are nondiffusive by TuTraSt analysis, and these are correctly predicted for 93 and 98% of the single-particle TuTraSt diffusion coefficients *D*_SP_ and multiparticle diffusion coefficients *D*_MP_, respectively.

**Figure 3:**
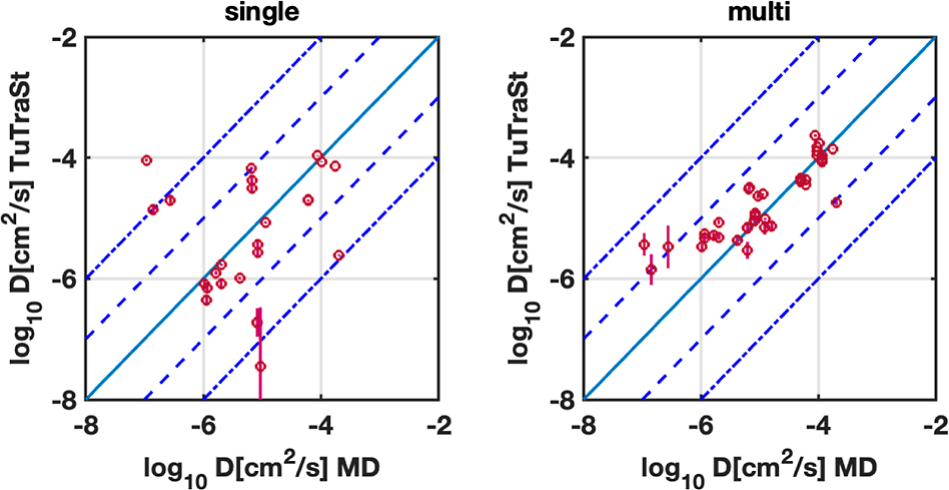
Validation set 1: Diffusion coefficients in the directions of the cell vectors are computed with the single-particle original TuTraSt algorithm (left) and multiparticle Ionic TuTraSt algorithm (right) on the *y*-axis relative to the corresponding diffusion coefficients computed with MD on the *x*-axis on a log–log scale. The dashed lines guide the limits for deviation of 1 and 2 orders of magnitude, respectively.

To validate the accuracy of the method prediction for diffusive structures, Figure 3 shows the comparison of the TuTraSt- and MD-obtained nonzero diffusion coefficients. Of the 42 directional diffusion coefficients computed by the MD, only 17 (40%) are correctly predicted by the single-particle model compared to 41 (98%) when adding the loading correction. Of the incorrectly predicted directional diffusion coefficients, 20 of the *D*_SP_ are false positives (i.e., incorrectly predicts that the ion can diffuse) and 20 are false negatives. In contrast, only six of the *D*_MP_ values are false positives and none are false negatives. Of these, one showed diffusion with MD, although 1.5 orders of magnitude lower than Ionic TuTraSt, thus being outside the accuracy limit, while the others showed no diffusion with MD. To gain a deeper understanding of why TuTraSt shows high diffusivity in two structures where MD predicts none, we analyze their individual diffusion mechanisms. The two cases show very different reasons for the overprediction. The first case (Li_4_GeO_4_) is caused by the fundamental differences in the sampling of the multiparticle PES by MD and MMC. When comparing these computed PESs, it is apparent that MMC samples are low-energy basins that are not accessible to MD due to a high energy barrier (∼70 kJ/mol). The presence of ions in these additional basins lowers energy barriers, resulting in the formation of diffusion channels at the edges of the cell (see Figure S3). The second case (Li_3_YBi_2_) shows, in contrast, very similar multiparticle PESs from both MD and MMC. Instead, the low diffusion predicted by MD can be rationalized by a correlated motion of ions through a limited number of admissible configurations as illustrated in Figure S4.

Although the existence of false positives decreases the overall efficiency of a potential screening study, it should be noted that these are less detrimental to the validations of the methodology compared to false negatives as they will not exclude potentially highly conductive materials.

**Figure 4:**
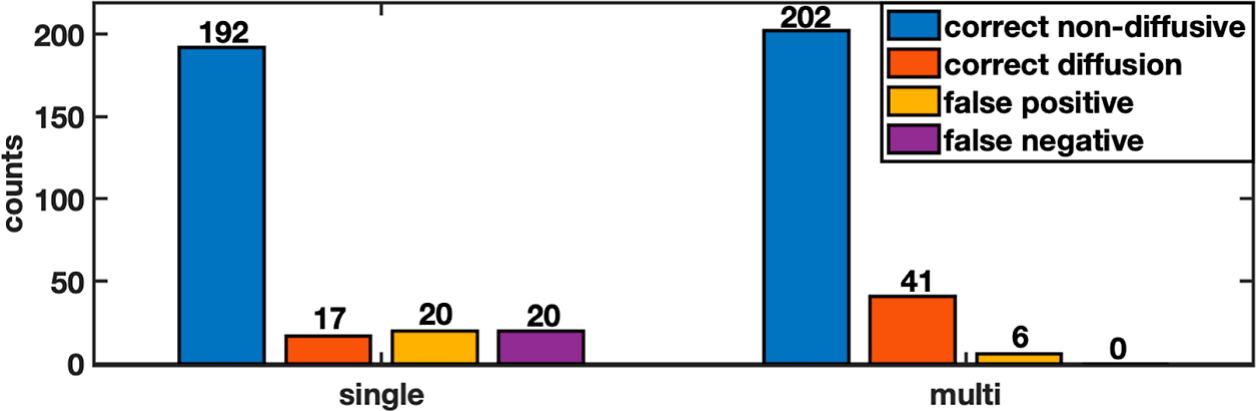
Bar diagrams showing the distributions of correctly (nondiffusive or diffusive) and incorrectly (false positives or false negatives) predicted diffusion coefficients at 1000 K by the single-particle TuTraSt method and multiparticle TuTraSt method, respectively.

Combining the results for diffusive and nondiffusive predictions, introducing the multiparticle correction shows a significant improvement in the correlation of TuTraST values with MD results as shown in the bar diagrams in Figure 4. The agreement increases from 82 to 98% with the correction, and the Spearman correlation coefficient increases from 0.42 to 0.87.

**Figure 5:**
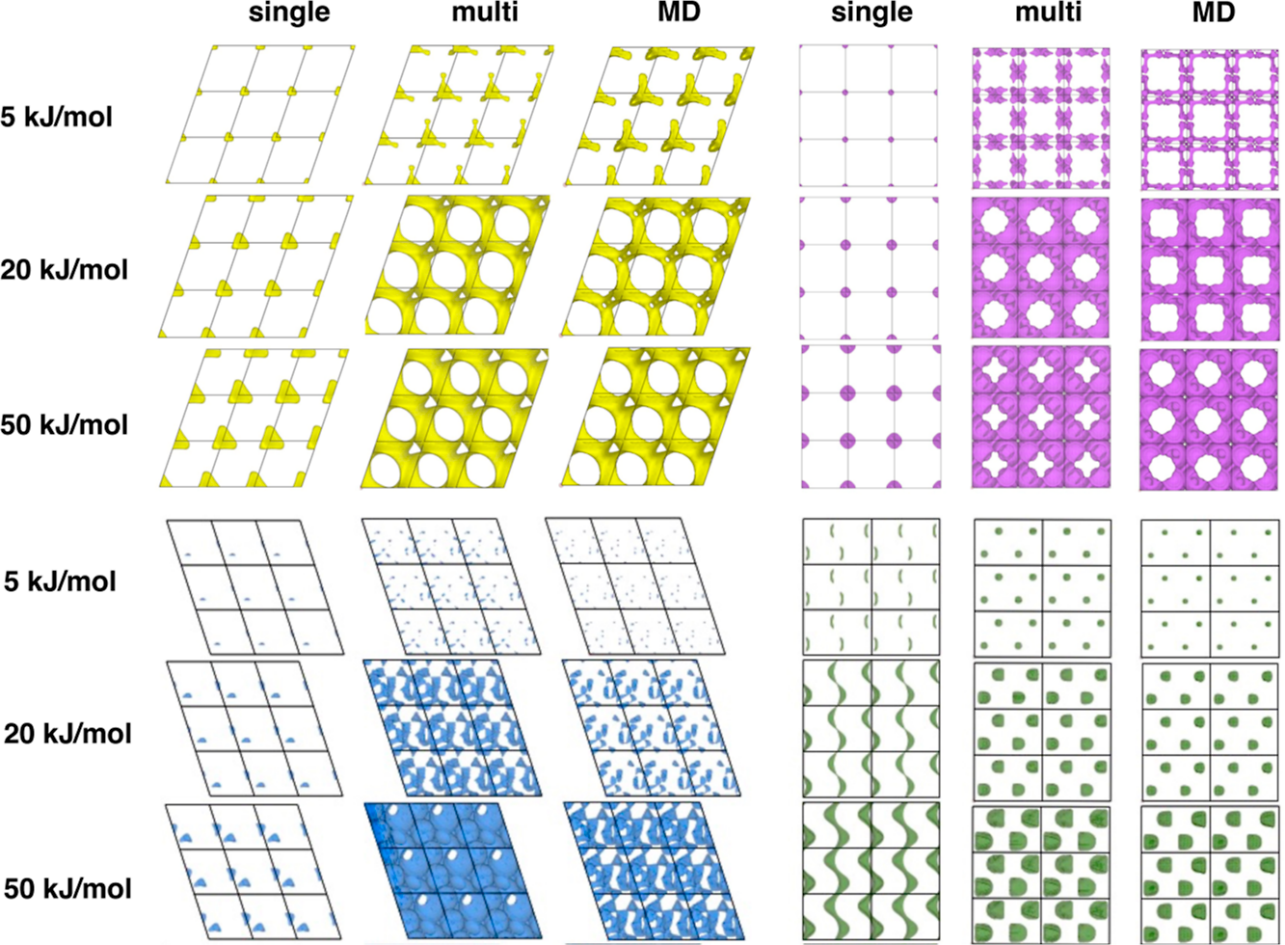
Potential energy isosurfaces from single-particle grids, multiparticle grids, and MD simulations for structures Li_2_CuSb (yellow), Li_8_Mg_4_Si_4_ (magenta), Li_8_TeN_2_ (blue), and Li_4_Cu_4_As_4_ (green). In the single-particle analysis, the first three structures are false negatives and the fourth structure is a false positive. The multiparticle grids correct the single-particle grids as seen by their good resemblance to MD PESs.

As is clear from the presented data, the improvements provided by the loading corrections introduced by the Ionic TuTraSt procedure are significant. It is instructive to further study the structures with the largest improvements individually. For a deeper understanding of the effect induced by the ion–ion interactions, we compare qualitative differences between the single-particle PES, the multiparticle PES, and the MD PES, as presented graphically in Figure 5 for four different structures and three different potential energy isovalues each. Each of these cases shows how the loading corrects the PES to agree with the MD PES qualitatively. In structures Li_2_CuSb, Li_8_Mg_4_Si_4_, and Li_8_TeN_2_, it is apparent that the energy barriers decrease for the Li diffusion when the ion–ion interaction is taken into account. Here, the structures go from nondiffusive in the single-particle PES case, which is shown by the unconnected isosurfaces at all energy isovalues, to highly diffusive in the multiparticle PES case, shown by the isosurfaces extending over the entire lengths of the unit cell. This is the most common effect observed. However, a few cases of the opposite are also observed, such as for structure Li_4_Cu_4_As_4_. Here, the energy barriers are instead increased upon loading, and the percolation channels seen in the single-particle PES are blocked off in the multiparticle case in accordance with the MD results.

**Figure 6:**
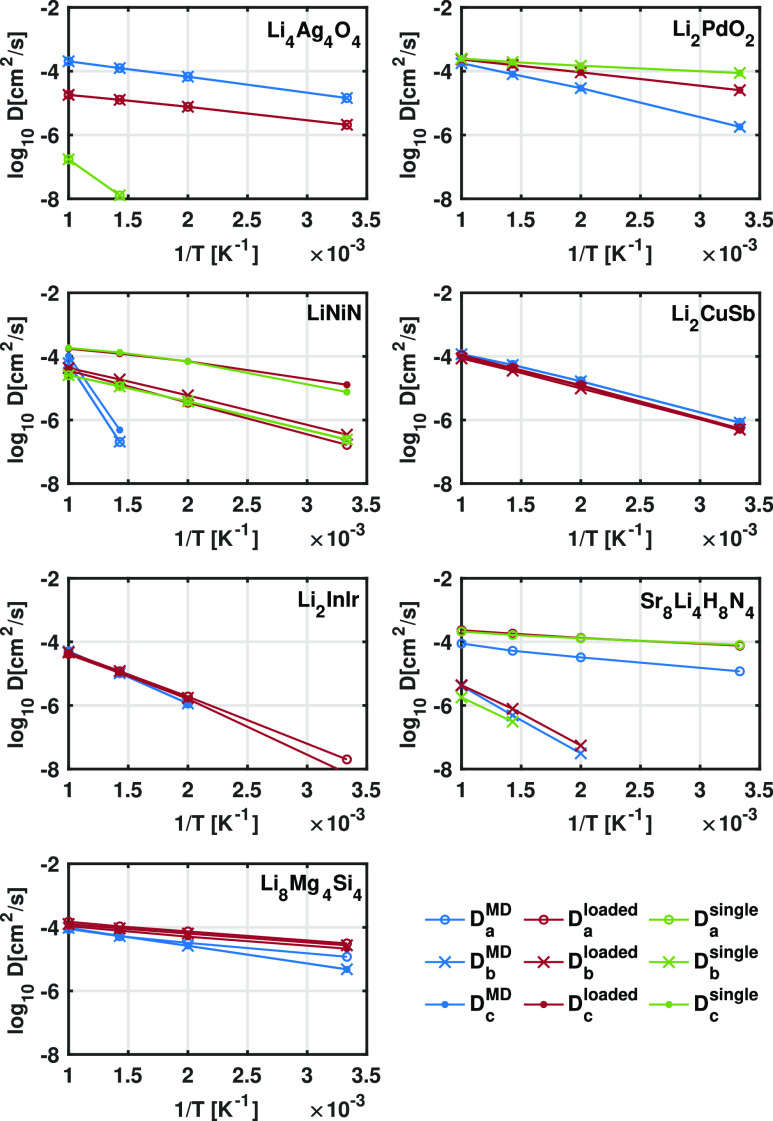
Arrhenius plots showing the temperature dependence of diffusion coefficients computed with the three different methods compared in this study for structures that Ionic TuTraSt predicts to be good ion conductors at room temperature. *D*_*a*_, *D*_*b*_, and *D*_*c*_ denote diffusion coefficients along the respective cell vector directions. Values are plotted as unfilled circles, crosses, and filled circles, respectively.

To validate the temperature dependence and to further validate the Ionic TuTraSt methodology’s ability to predict structures with high ion diffusivity at room temperature, Arrhenius plots are presented in Figure 6. From the Ionic TuTraSt approach, seven structures were predicted to be highly diffusive at 300 K (i.e., *D*_MP_ > 10^–6^ cm^2^/s). Of these, the MD simulations show similar Arrhenius slopes and confirmed the high diffusivity all the way down to 300 K in six cases. In four of the cases (Li_4_Ag_4_O_4_, Li_2_CuSb, Li_2_InIr, and Li_8_Mg_4_Si_4_), the agreement with MD Arrhenius profiles is significantly improved with the implemented Ionic TuTraSt loading correction where the single-particle TuTraSt predicts several orders of magnitude lower or no diffusion. In two cases (Li_2_PdO_2_ and Sr_8_Li_4_H_8_N_4_), the loading correction shows little effect on the diffusion coefficients. Thus, both the single- and multiple-particle TuTraSt calculations agree with the MD Arrhenius behavior.

In the final case (LiNiN), the Arrhenius behavior, however, deviates significantly. Although the agreement at 1000 K between all methods is excellent, the MD shows a more negative slope, reaching a *D*_MD_ value of < 10^–6^ cm^2^/s already at 700 K. When further analyzing the PES constructed from the full simulation cell from the 1000 and 700 K MD trajectories, respectively, a long-range ordering creating discontinuities in the diffusion channels is identified at 700 K (see Figure S6), which is not present at 1000 K. These interesting results indicate that there exist long-range energy barriers related to the structural ordering of the Li that extend beyond the length of the unit cell, which cannot be overcome at lower temperatures.

### Validation Set 2: Pinball Grids

**Figure 7:**
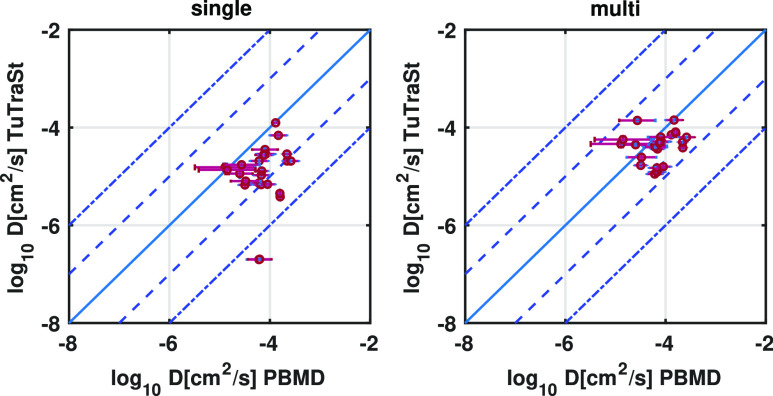
Validation set 2: Directional diffusion coefficients computed with the single-particle original TuTraSt algorithm (left) and multiparticle Ionic TuTraSt algorithm (right) on the *y*-axis relative to the corresponding diffusion coefficients computed with MD on the *x*-axis on a log–log scale. The dashed lines guide the limits for deviation of 1 and 2 orders of magnitude, respectively.

For the nine structures (27 directional diffusion coefficients) in validation set 2, Ionic TuTraSt also shows excellent performance. As shown in Figure 7, all of the *D*_MP_ values are predicted within 1 order of magnitude of the diffusion coefficients computed with the DFT-based Pinball-MD method, *D*_PBMD_.^10^

**Figure 8:**
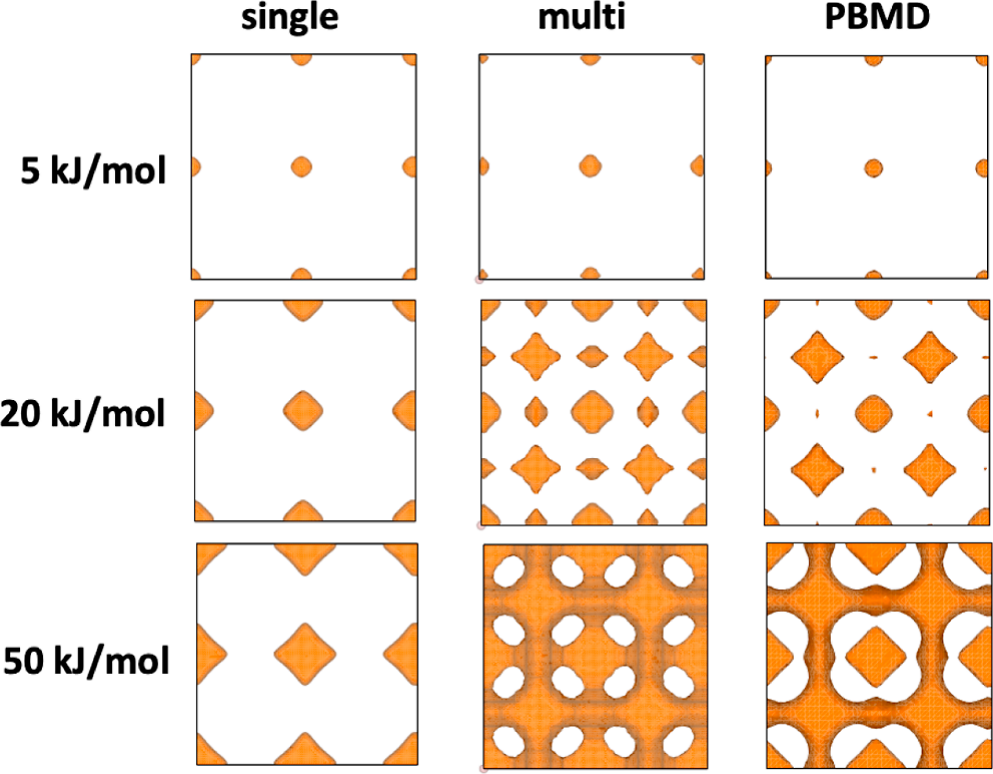
Comparison of PES isosurfaces for Li_8_Cs_4_I_12_. The multiparticle PES shows the formation of a secondary diffusion channel in agreement with the PBMD PES, which is not present in the single-particle PES.

The single-particle TuTraSt prediction produces four false negative diffusion coefficients, in two directions, in two different structures. In all of these cases, the Ionic TuTraSt loading correction adjusts the results to the accepted accuracy. Of these two structures, Li_8_Cs_4_I_12_ deserves special attention as it exemplifies, in a concrete manner, how the loading can have a very substantial effect. The comparison of PES isosurfaces for this structure presented in Figure 8 makes clearly visible how the loading correction provides a remarkable improvement of the overall agreement with the MD PES, in turn, improving the accuracy of the predicted diffusion coefficients significantly. In the single-particle PES, four basins are observed in the unit cell, and the energy barriers between them are high, ∼ 80 kJ/mol. However, the stoichiometry of this structure is eight Li per unit cell. When loading the Li, the first four Li are situated in the deep basins and can effectively be considered immobile parts of the framework lattice. The remaining four Li then experience a secondary PES arising at ∼10 kJ/mol relative to the immobile Li. This secondary PES forms diffusion channels with much lower energy barriers ∼25 kJ/mol, enabling high diffusivity.

### Computational Efficiency

To focus the discussion of computational efficiency on the method rather than software choices, parallelization and HPC, implementation, and efficiency, we carry out an order analysis of the Ewald summation, as this constitutes the majority of the computational workload for both the MD and MC simulations as well as for the grid calculations. The standard Ewald summation, as implemented in LAMMPS, has an  dependency on the number of interacting particles *N*_part_ in the simulated system.^27^ For the MD simulations, the number of interacting particles consists of all atoms in the system, both Li (*N*_Li_) and lattice framework atoms (*N*_FW_), thus *N*_part_ = *N*_Li_ + *N*_FW_. For the loading correction MC simulation, *N*_part_ = *N*_Li_ since only Li particles interact. For the grid sampling, a single Li samples the framework lattice, thus *N*_part_ = *N*_FW_ + 1. Furthermore, the computational cost scales linearly with the number of simulation steps, which, for the MD simulations, correspond to the number of time steps *N*_step_^MD^, for the MC simulations, to the product of the number cycles *N*_cycle_^MC^ with the number of Li particles *N*_Li_, and for the single-particle grid construction, to the number of grid points *N*_grid_. This results in scaling orders of *S*_MD_, *S*_MC_, and *S*_grid_ for the MD, MC, and grid sampling calculations, respectively.
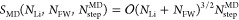

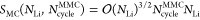

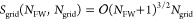


The scaling order of the full Ionic TuTraSt workflow is thus given by
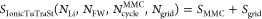


**Figure 9:**
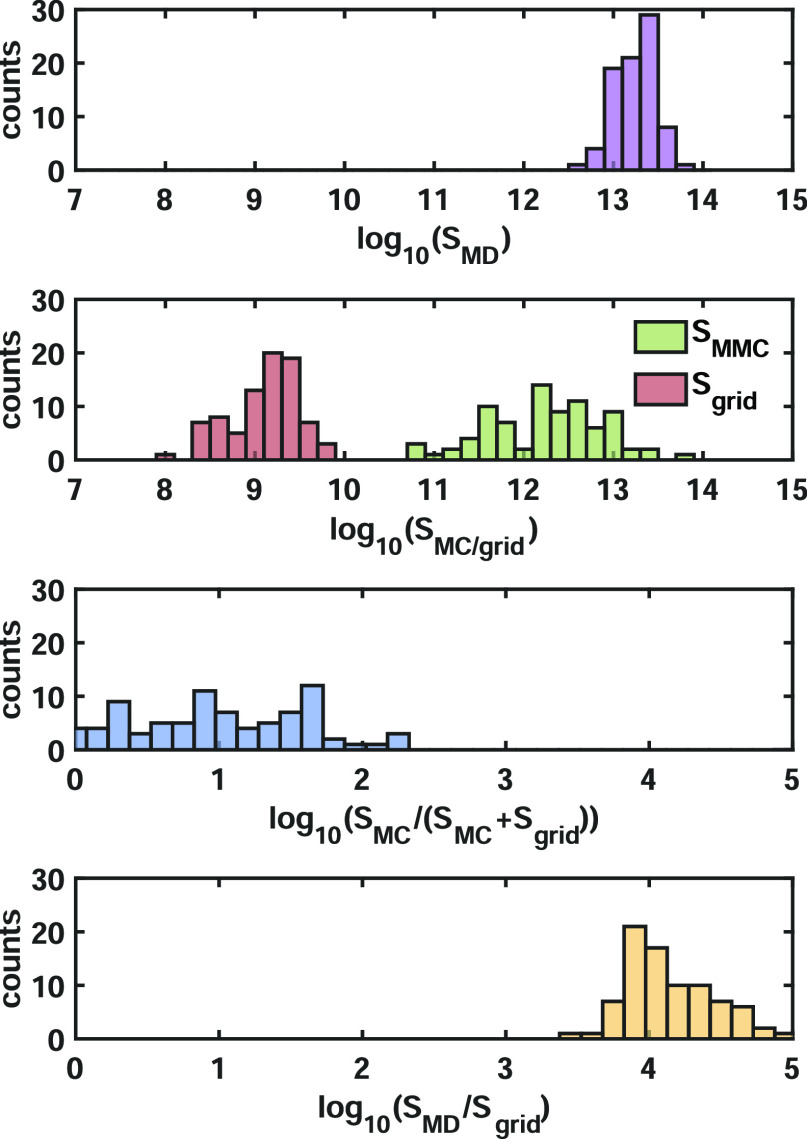
Comparing the computational efficiency: The Ionic TuTraSt procedure shows a computational efficiency speed-up ranging up to more than 2 orders of magnitude, compared to MD, when identical pair potential functions are used in the methods compared (third panel). When the single-particle grid calculation costs dominate over the MMC routine, this speed-up reaches 3 to 5 orders of magnitude (fourth panel).

The efficiency between the MD and Ionic TuTraSt routines of validation set 1 is compared in Figure 9 that shows the respective scaling factors per structure. Note that in the study of validation set 1, identical pair potential functions are used for both methods, therefore allowing for their direct comparison.

The computational speed-up ratio of the Ionic TuTraSt routine compared to MD for validation set 1 ranges up to 2.5 orders of magnitude with an average speed-up ratio of 25. The overall speed-up of Ionic TuTraSt compared to MD simulation is enabled by the MMC routine only computing the Li–Li interactions explicitly at each step while taking Li–framework interactions from the single-particle grid. In addition, the topological analysis on the unit cell permits an averaging of the sampling over all unit cells of the supercell in the simulation, which allows for a lower number of overall MMC simulation steps compared to MD.

Figure S7 shows that the Ionic TuTraSt speed-up has a strong inverse dependence on the Li ratio in the structure, where one of the structures with the highest Li ratio [*N*_Li_/(*N*_Li_ + *N*_FW_) = 0.66] instead shows a decreased efficiency by half compared to MD.

From the comparison of the force-field-based calculations carried out on validation set 1, the speed-up can be considered significant. The expected speed-up for DFT-based potential energy calculations compared to DFT MD has the potential to be even much larger. As shown in the case study using validation set 2, the Pinball-MD results are reproduced to good accuracy by the Ionic TuTraSt routine, although Ionic TuTraSt decouples the generation of the single-particle PES from the computation of the Li–Li interaction energies, which is done with cheaper classical force field interactions. Compared to classical MD simulations, Ionic TuTraSt saves computational costs mainly in generating the single-particle PES. In contrast, it is the computation of the Li–Li interaction energies that is responsible for the difference in the computational cost of Ionic TuTraSt compared to that of DFT-based methods, with DFT-based interactions being orders of magnitude more computationally demanding for each single energy calculation. Thus, for the efficiency analysis comparing DFT-based MD with DFT-based Ionic TuTraSt, it is sufficient to compare only the scaling of the grid sampling calculation. If the structures of validation set 1 were simulated using a DFT method, the speed-up from Ionic TuTraSt would be as high as 3–5 orders of magnitude.

It is also worth noting that in the case where diffusion coefficients at several different temperatures are to be predicted, Ionic-TuTraSt only requires the computation of one PES grid, which is valid at all temperatures, since the PES grid is temperature-independent, while for MD, each temperature requires a separate simulation.

### Conclusions and Outlook

With Ionic TuTraSt, we present a method to predict the diffusion of strongly interacting particles within a rigid molecular framework. This approach is based on constructing and analyzing the geometry and topology of the PES felt by the migrating particles. The multiparticle PES is constructed from an MMC routine that samples the particle configurations based on a potential energy that is estimated as the sum of a precalculated particle-framework component and a component describing the classical interaction of the mobile particles. This provides a multiparticle correction to the previously developed TuTraSt algorithm, which is an automated workflow to compute diffusion coefficients using a kMC simulation based on the geometric and topological identification of positions and the heights and depths of transition states and energy basins, respectively.

Being based on the analysis of the potential energy only brings benefits in terms of efficiency and automatization compared to state-of-the-art methods, i.e., MD and NEB, to model rare-event diffusion processes. The analysis allows for average sampling probabilities over repeated units in the multiparticle MMC routine, which improves the efficiency compared to MD that depends on trajectories extending over longer length scales. Furthermore, using kMC produces particle trajectories at a very low computational cost even for very slow diffusers that require long time-scales to reach the diffusive regime. Slow diffusion is a limiting factor for brute-force MD simulations. An additional benefit of our analysis of the geometry and topology of potential energies lies in its independence from prior knowledge of diffusion pathways, which is required to apply, e.g., NEB methods, thermodynamic integration, or metadynamics. This independence allows for automatization.

In a case study of Li diffusion in crystalline inorganic SSE candidates, we validated our method against classical MD and DFT-based Pinball MD. We found that the diffusion coefficients were predicted to a high accuracy: 98% is predicted within 1 order of magnitude from MD. Our efficiency analysis based on the number of pair-potential calculations required shows that the Ionic TuTraSt approach provides a speed-up of up to 3 orders of magnitude compared to MD when using classical force fields and up to 5 orders of magnitude when using higher-level energy calculation methods.

The Ionic TuTraSt approach allows for diffusion calculations on all levels of theory depending on the method used to generate the single-particle potential energy. This makes it a promising framework to predict diffusion coefficients at the DFT level at a low computational cost. The MMC routine that produces the potential that accounts for interactions between migrating particles is computed using classical interactions. It thus adds a negligible computational cost to the workflow when using single-particle potentials obtained from higher levels of theory calculations, e.g., DFT. In this case, the limiting factor of the workflow is the cost of producing the single-particle grid. In a parallel study, we are therefore developing strategies to reduce the number of grid points for which energy values need to be calculated when constructing the single-particle grid. To this end, the symmetry of crystalline materials can be exploited. Additionally, strategies can also be employed that exclude energetically or sterically inaccessible volumes or utilize interpolation functions. Combined, these strategies enable the construction of full PES grids from only a few hundred single-point energy calculations. This will further increase the Ionic TuTraSt speed-up significantly and make high-throughput screening of ion diffusion of potential SSE materials at the density functional level of theory truly feasible.

The Ionic TuTraSt methodology provides a framework to predict the diffusion of interacting particles not only for SSEs but also for any system where rare-event processes can describe the diffusion of mobile particles within a rigid structural framework. This includes membrane materials for gas separation and water purification. To further extend the applicability of the methodology, future developments are considered, such as (1) strategies to include effects of lattice vibrations (phonons) for systems where phonon effects have a significant impact on the diffusion, (2) strategies to treat systems where the framework lattice cannot be considered spatially defined, (i.e., soft materials such as polymer electrolytes), and (3) strategies to treat mixtures of different types of mobile particles that interact with each other.

## Supporting Information Available

Additional computational details and analysis results (PDF)
Data files containing numerical values of diffusion coefficients used in the plots (ZIP)
