# Supplementary material for: Predicting Ion Diffusion from the Shape of Potential Energy Landscapes
Source: J Chem Theory Comput. 2023 Dec 19;20(1):18–29. doi: 10.1021/acs.jctc.3c01005 (PMC10782449; doi:10.1021/acs.jctc.3c01005)
Supplement: Supplementary file 1 — ct3c01005_si_001.pdf [file ct3c01005_si_001.pdf]

Supporting Information: Predicting ion diffusion from the shape of potential energy landscapes

Hannes Gustafsson,[†] Melania Kozdra,[†] Berend Smit,[‡] Senja Barthel,[¶] and Amber Mace[*,†]

†*Department of Chemistry – Ångström, Uppsala university, SE-751 21 Uppsala, Sweden*

‡*Institut des Sciences et Ingénierie Chimiques, Valais, Ecole Polytechnique Fédérale de Lausanne (EPFL), Rue de l'Industrie 17, CH-1951 Sion, Switzerland*

¶*Department of Mathematics, Vrije university, 1081 HV Amsterdam, Netherlands*

E-mail: amber.mace@kemi.uu.se

DFT calculation of REPEAT partial charges

In order to obtain the electrostatic potentials used to calculate partial charges with the REPEAT method, single point calculations were performed on a unit cell of each structure using density functional theory (DFT) with the Perdew-Burke-Ernzerhof (PBE)[S1] functional. Both DFT calculations and the succeeding[S2] analysis were performed using the CP2K package.[S3] Kohn-Sham orbitals were expanded using double-ζ shorter-range (DZVP-MOLOPT-SR-GTH) basis sets.[S4] Godecker-Tetter-Hutter[S5] norm-conserving pseudo potentials were used in order to account for the nuclear cores. Periodic boundary conditions were applied in every dimension. Furthermore, a real cutoff and planewave cutoff were equal to 50 Ry and 400 Ry respectively whilst Gaussians were mapped onto five grid levels. The Conjugate Gradients minimizer was used while the accuracy of an SCF cycle was kept at the level of $5 \cdot 10^{-6}$. The resulting partial charges for each structure in

validation set 1 are presented in table S1.

Table S1: Materials Cloud structure code, chemical formula and computed REPEAT charges given for each atom type in elementary charge units for structures in validation set 1.

Structure code	Chemical formula	Partial charges
54189	Li_2ZnGe	Li 0.72, Zn 1.5, Ge -2.95
54209	$LiNbO_3$	Li 0.92, Nb 2.59, O -1.17
54297	LiAgO	Li 0.14, Ag -0.015, O -0.09
54449	Li_2AlIr_2	Li 1.25, Al -5.29, Ir 1.39
54683	Li_2PtO_6	Li 0.72, Pt 1.47, O -0.48
54837	Li_2HfF_6	Li 0.92, Hf 2.28, F -0.69
54865	Li_2PdO_2	Li 0.16, Pd -0.53, O 0.11
54879	Li_3YBi_2	Li 0.98, Y 2.32, Bi -2.63
54884	$Rb_2LiScCl_6$	Rb 0.78, Li 0.95, Sc 1.34, Cl -0.64
55184	$LiSc(TlCl_3)_2$	Li 0.90, Sc -0.49, Tl 0.38, Cl -0.49
55319	LiNiN	Li 0.59, Ni 0.03, N -0.63
55983	LiAuS	Li 0.74, Au 0.28, S -1.02
56568	Li_2LaGe	Li 0.91, La 1.01, Ge -2.83
56627	Li_2CoSb	Li 0.79, Co 0.37, Sb -1.94
57347	$LiAlH_4$	Li 0.96, Al 1.34, H -0.58
57382	RbLiSe	Rb 0.61, Li 0.63, Se -1.25
57448	Li_2ZrN_2	Li 1.0, Zr 2.8, N -2.39
57608	Li_2HfCdF_8	Li 1.03, Hf 2.67, Cd 1.7, F -0.8
57644	LiBeAs	Li 0.84, Be 0.71, As -1.55
57761	$LiAlH_4$	Li 0.82, Al 1.47, H -0.57
59631	Li_2CuSb	Li 0.7, Cu 0.94, Sb -2.35
59632	$LiInSe_2$	Li 0.69, In 0.74, Se -0.72
59715	LiInSn	Li 0.9, In -1.41, Sn, 0.52
59948	Li_3H_4Rh	Li 0.45, H -0.21, Rh -0.53

60450	LiBeP	Li 0.41, Be 0.55, P -0.96
61111	$NaLiCO_3$	Na 0.81, Li 0.73, C 1.3, O -0.95
61237	$LiZnPS_4$	Li 0.65, Zn 0.77, P 0.36, S -0.45
61329	$CsLi_2(HO)_3$	Cs 0.71, Li 0.64, H 0.21, O -0.87
63257	$LiCaAlN_2$	Li 0.71, Ca 1.20, Al 1.48, N -1.69
63279	$LiMgVO_4$	Li 0.88, Mg 1.84, V 1.93, O -1.16
63600	$LiGeTe_2$	Li 0.56, Ge -0.01, Te -0.28
63663	$LiCdCoF_6$	Li 0.76, Cd 1.49, Co 0.86, F -0.52
63922	LiSnAu	Li 0.81, Sn -1.11, Au 0.30
64383	$Sr(Li_2P)_2$	Sr 0.98, Li 0.48, P -1.44
64396	$Ba_3(LiBi)_4$	Ba 1.29, Li 0.69, Bi -1.66
64754	Li_2InIr	Li 0.74, In -2.91, Ir 1.43
64867	$LiGaO_2$	Li 0.84, Ga 1.32, O 1.08
65350	Rb_2LiYCl_6	Rb 0.78, Li 1.04, Y 1.57, Cl -0.7
65898	$Cs_2Li_2TiO_4$	Cs 0.82, Li 0.84, Ti 1.66, O -1.25
66685	$LiLuF_4$	Li 1.03, Lu 2.31 F -0.83
66876	$KLiMoO_4$	K 0.89, Li 1.28, Mo 2.34, O -1.13
66985	$LiYF_4$	Li 1.06, Y 2.44, F -0.88
67415	$CsLi(H_2N)_2$	Cs 0.59, Li 0.83, H 0.32, N -1.34
67418	$Li_4Ca_3(SiN_3)_2$	Li 0.83, Ca 1.33, Si 1.86, N -1.84
67779	$LiCaAlF_6$	Li 0.77, Ca 1.63, Al 0.89, F -0.55
67785	Li_3PS_4	Li 0.79, P 0.95, S -0.83
67806	$LiNbWO_6$	Li 0.82, Nb 2.58, W 1.86, O -0.88
67999	$KLiWO_4$	K 0.88, Li 1.29, W 2.41, O -1.15
68042	LiMgN	Li 1.42, Mg 2.07, N -3.34
68103	$Sr_3Li_4(GeN_3)_2$	Sr 1.47, Li 0.62, Ge 1.66, N -1.89
68120	$LiMoS_2$	Li 0.62, Mo 0.7, S -0.66
68242	$LiSc(SiO_3)_2$	Li 0.84, Sc 1.62, Si 1.37, O -0.87
68674	$LiLa_2SbO_6$	Li 1.21, La 2.38, Sb 2.45, O -1.4

68894	$LiSbO_3$	Li 0.95, Sb 2.41, O -1.12
68960	Na_2LiAlH_6	Na 0.89, Li 0.85, Al 0.39, H -0.5
69116	$LiYSe_2$	Li 0.79, Y 0.92, Se -0.85
69642	$LiIn(SiO_3)_2$	Li 0.86, In 1.37, Si 1.53, O -0.88
70003	LiCSN	Li 0.84, C 0.54, S -0.40, N -0.98
70442	Sr_2LiReO_6	Sr 1.69, Li 2.07, Re 3.26, O -1.45
70681	$LiHSeO_3$	Li 0.87, H 0.41, Se 0.53, O -0.6
71368	$LiYF_2$	Li 0.59, Y 0.41, F -0.5
71681	$LiSc(WO_4)_2$	Li 1.02, Sc 2.16, W 2.38, O -0.99
71925	$LiLu(WO_4)_2$	Li 1.05, Lu 2.02, W 2.28, O -0.95
71990	Sr_2LiH_2N	Sr 0.49, Li 0.18, H -0.31, N -0.55
72018	Cs_2LiYCl_6	Cs 0.68, Li 0.91, Y 1.48, Cl -0.62
72372	LiLuGe	Li 0.77, Lu 1.35, Ge -2.12
72631	Li_2SiS_3	Li 0.67, Si 0.38, S -0.58
72652	$Li_2Ge_2O_5$	Li 0.98, Ge 1.47, O -0.98
73298	$CsLiBr_2$	Cs 1.19, Li 0.96, Br -1.07
73679	Li_2AlRh_2	Li 1.23, Al -4.11, Rh 0.82
73766	LiCaAs	Li 0.84, Ca 1.32, As -2.16
74395	Li_4GeO_4	Li 0.85, Ge 1.27, O -1.17
74865	$LiAlSiO_4$	Li 0.91, Al 1.67, Si 1.8, O -1.09
75073	Li_2CdSiO_4	Li 0.86, Cd 1.34, Si 1.31, O -1.10
75138	Li_2MgSi	Li 0.62, Mg 0.96, Si -2.20
75630	SrLiSb	Sr 1.36, Li 0.86, Sb -2.22
75961	Li_8TeN_2	Li 0.64, Te -1.40, N -1.86
76276	$LiCaFeF_6$	Li 0.82, Ca 1.62, Fe 1.07, F -0.59
76595	Li_2ZrF_6	Li 0.92, Zr 2.31, F -0.69
77893	$LiScP_2O_7$	Li 0.91, Sc 1.94, P 1.64, O -0.88
77899	$LiYMo_3O_8$	Li 0.78, Y 2.14, Mo 1.43, O -0.9
78086	$LiNbSe_2$	Li 0.53, Nb 0.57, Se -0.55

78355	$LiCaCrF_6$	Li 0.8, Ca 1,62, Cr 1.20, F -0.60

Choice of single-particle grid sizes

The multi-particle MMC routine in the Ionic TuTraSt workflow was run both with a 0.1 Å × 0.1 Å × 0.1 Å and 0.2 Å × 0.2 Å × 0.2 Å single-particle grid as input to test the effect of the grid size resolution on the stability and accuracy. Figure S1 shows that the accuracy of the the prediction of diffusion coefficients compared with MD does not decrease when the single-particle grid resolution decreases from 0.1 Å to 0.2 Å. Rather, the finer grid resolution shows a lower stability with three false positives as the Li-counts are spread over a larger number of volumetric bins, thus rendering more noise in the statistics and a less smooth PES surface compared to the 0.2 Å grids.

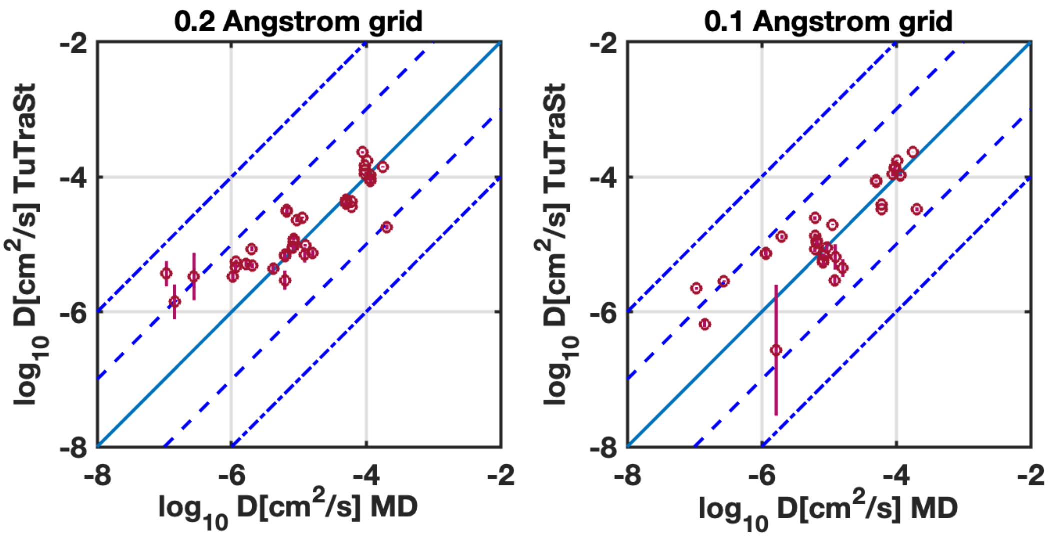

Figure S1: Directional diffusion coefficients for validation set 1 computed with single-particle grid sizes 0.2 Å (left) 0.1 Å (right) on the y-axis relative to the corresponding diffusion coefficients computed with MD on the x-axis on a log–log scale. The dashed lines guide the limits for deviation of one and two orders of magnitude, respectively.

Effect of Li partial charge for validation set 2

Different partial charges were tested for Li during the multi-particle MMC simulations; $q = 0.25e$, $0.5e$, $0.75e$ and $1e$, as well as Li charges computed by the REPEAT method as

described in the previous section. To validate which partial charge best reproduces the MD results, both the directional diffusion coefficients and the break-through energies are compared. The break-through energy is the lowest energy at which a percolation channel in a given direction appears in the PES.

The results presented in figure S2 show that both diffusion coefficients and break-through energies are best reproduced by partial charges $q = 0.25e$, and $0.5e$, while the use of higher charges and REPEAT produces false negatives. When comparing the deviations of break-through energies, $q = 0.5e$ performs slightly better than $q = 0.25e$ with mean absolute deviations of 4 and 5.8 kJ/mol, respectively.

False positive diffusion predictions

The performed validation studies show three different reasons of false positive predictions by the Ioniq TuTraSt methods compared to MD simulations. Their analysis is instructive in order to understand the limits of the method and to provide insight for future development.

To explain the false positive result predicted by Ionic TuTraST for Li_4GeO_4 predicted at 1000 K, we compare the single-particle, multi-particle and MD-generated PES shown in Figure S3. The single-particle PES shows four energy basins centered in the unit cell along the z-axis with energy barriers > 70 kJ/mol (primary PES region). The multi-particle PES shows additional low energy regions (secondary PES region) caused by the interacting eight stoichiometric Li of the unit cell. These secondary regions form diffusion channels in all directions at energies < 60 kJ/mol and the structure is predicted as highly diffusive by Ionic TuTraSt. The MD, on the other hand shows a non-diffusive structure where the low energy basins within the secondary region are completely isolated from each other. In addition, the primary PES region is not present at all. The large differences between the multi-particle and MD-generated PES is rationalized by the methodological difference in the sampling between MD and MMC. In the MD simulation the probability of entering the primary region is very low as the energy barriers to reach it is high. For

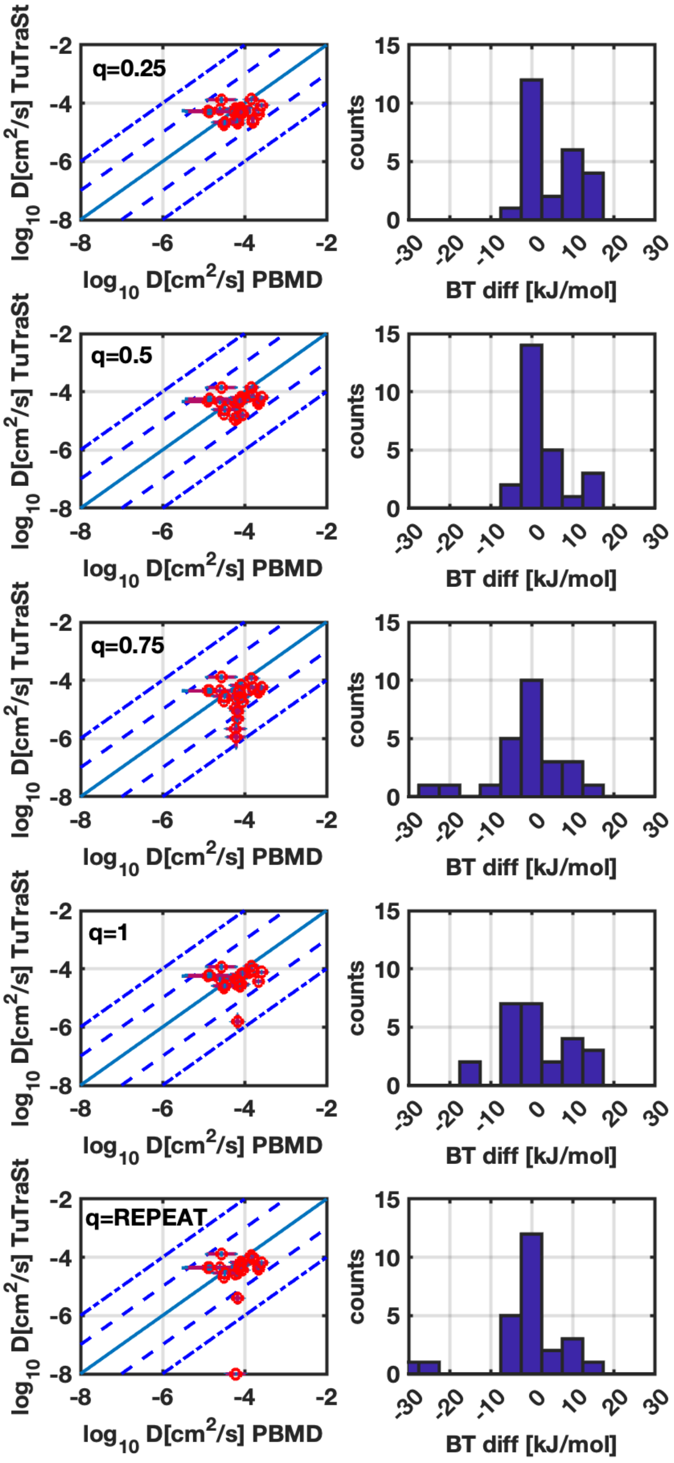

Figure S2: Comparison of Ionic TuTraSt performance using different charges (q) for the Li during the multi-particle MMC simulation for validation set 2. Plots on the left show diffusion coefficients obtained from Ionic TuTraSt against from diffusion coefficients obtained from PBMD simulations. The dashed lines guide the limits for deviation of one and two orders of magnitude, respectively. Histograms on the right show the difference in break-through (BT) energies obtained by Ionic TuTraSt relative to to PBMD.

MMC, on the other hand, the probability of sampling a configuration depends only on its configurational energy, and not on the path that reaches it. Thus MMC samples the primary regions. In turn, it appears that in the multi-particle grid, the secondary PES region is strongly affected by the presence of Li in the primary PES region, resulting in a significant decrease of the energy barriers and the formation of diffusion channels.

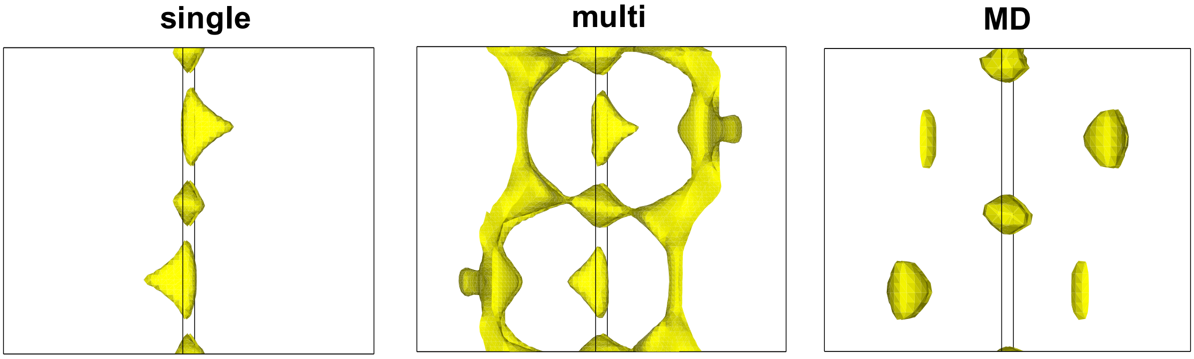

Figure S3: Potential energy isosurfaces at 60 kJ/mol from single particle grids, multi-particle grids, and MD simulations for structure Li_4GeO_4 are shown.

The reason for Li_3YBi_2 to be predicted as highly diffusive diffusive by Ionic TuTraSt but not by MD lies in the particular spatial arrangement of the basins. Although the multi-particle PES from both MD and MMC are very similar, Ionic TuTraSt and MD simulations describe different mechanisms. The radial distribution function of the MD reveals that neighbouring Li ions do not come closer than 2 Å while the probability peaks at about 2.5 Å, which corresponds to $\sim 1/2$ cell parameter in the x and y-directions. But the PES shows several basins of closer distance that the MMC permits to be occupied simultaneously. It appears that the unit cell of this structure has many more basins than ions but those basins can only be occupied in some configurations that respect a minimum distance between the ions. This constraint and the resulting correlated motion are not accounted for by the kMC simulation. Consequently Ionic TuTraSt overpredicts the diffusion coefficients.

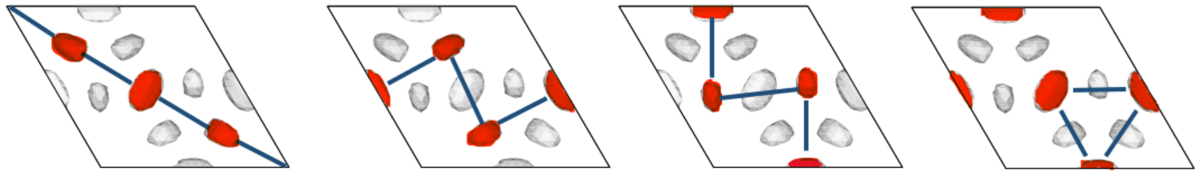

Figure S4: Potential energy isosurfaces in the xy-plane at 20 kJ/mol from MD simulations for structure Li_3YBi_2 (in white). The configurations that the three stoichiometric Li ions can take in the unit cell are presented in red. The radial distribution function of the MD has a first peak at around 2.5 Å, which is the approximate lengths between the sides occupied in the shown configurations (red), suggesting that it is highly improbable that sides with lower distance between them are simultaneously occupied.

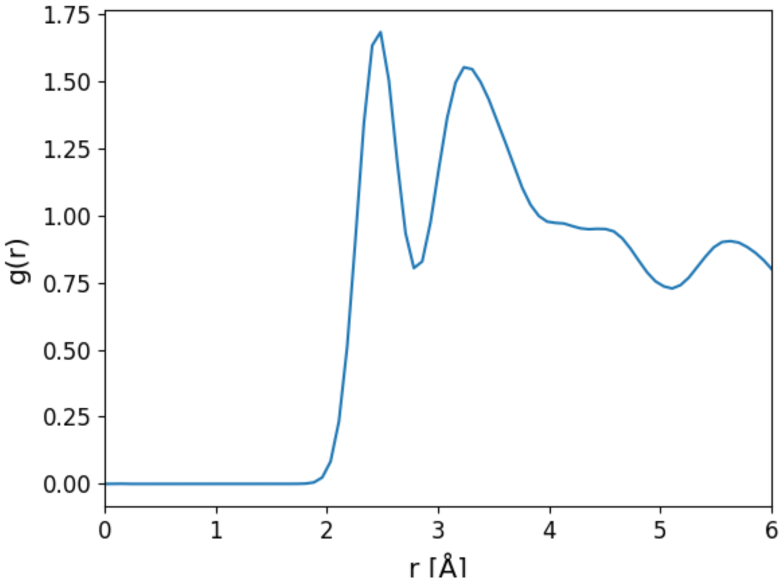

Figure S5: The Li−Li radial distribution function in Li_3YBi_2 computed from MD simulations.

In the third case, for the LiNiN structure, the false positive result is observed from the Arrhenius plot (Figure 6) at lower temperatures. At 1000 K the agreement is accurate, however from 700 K and below, a long range ordering in the x-direction emerges that extends across the full simulation. This long-range ordering breaks the symmetry and the basins do not merge into percolated diffusion channels. This effect is not seen in the multi-particle PES as the MMC simulation does not get stuck in local minima and can sample all low energy states more efficiently. Also, when constructing the multi-particle PES the Li sampling counts are averaged over all unit cells to a single unit cell PES, and thus possible long-range effects are not detected.

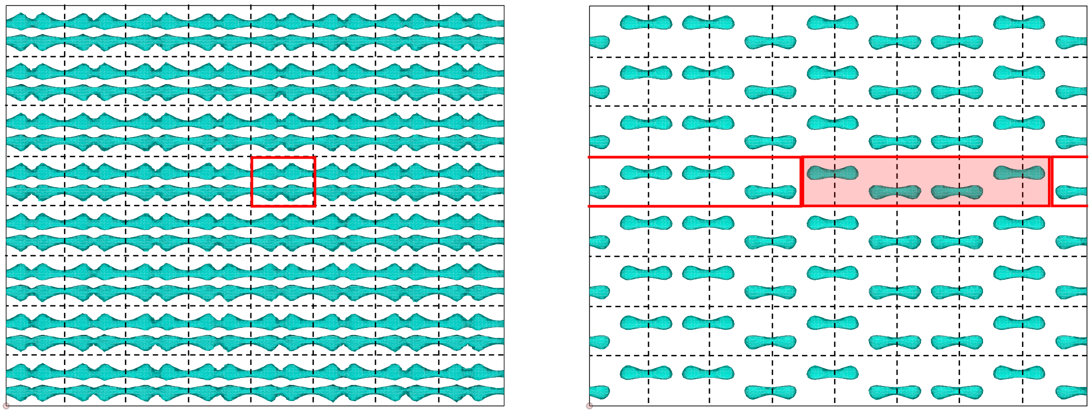

Figure S6: The MD PES isosurface (20 kJ/mol) of LiNiN from the 700 K simulation (right) shows a long-range Li^+ ordering compared to 1000 K (left). The dashed lines show the 8×8 unit cell boxes building up the simulation cell in the xy plane. The red boxes show the repeating unit for the respective temperature. At 1000 K, the repeating unit corresponds to the unit cell. In contrast, at 700 K, the periodic unit in the x-direction extends across the whole simulation cell, consisting of two inverted blocks with four unit cells each (shaded and transparent boxes, respectively).

Efficiency dependencies

Figure S7 shows how the speed-up ratios (as defined by the order analysis), of the respective single-particle grid and multi-particle MMC routines vs MD sampling, depend on the stoichometric Li-ratio and the number of grid points. Here, the dependencies are presented for different scaling orders; $\mathcal{O}(N^2)$ for direct summation of the Ewald method, $\mathcal{O}(N^{3/2})$ for standard Ewald summation as implemented in LAMMPS (used in this study) and $\mathcal{O}(N \log N)$ corresponding to the particle-particle particle-mesh (PPPM) method.[S6]

When using classical force-fields for the both single-particle grid sampling as well as the multi-particle MMC routine, the computational cost of the MMC routine dominates over the single-particle grid construction. The order of speed-up of Ionic TuTraSt over MD can be estimated by $\log_{10}(S_{MD}/S_{MMC})$ such as in the left panel of figure S7. From this plot it is clear the speed-up efficiency is strongly dependent on the Li-ratio of the structure. When a method with significantly higher computational cost (i.e. DFT) is used for the grid-sampling, the sampling routine becomes the dominating cost and the order of speed-up of and the Ionic TuTraSt over MD can instead be estimated by $\log_{10}(S_{MD}/S_{grid})$

as shown in the right panel of figure S7. In this case the speed-up efficiency is strongly dependent on the number of grid points, which are dependent on the unit cell parameters.

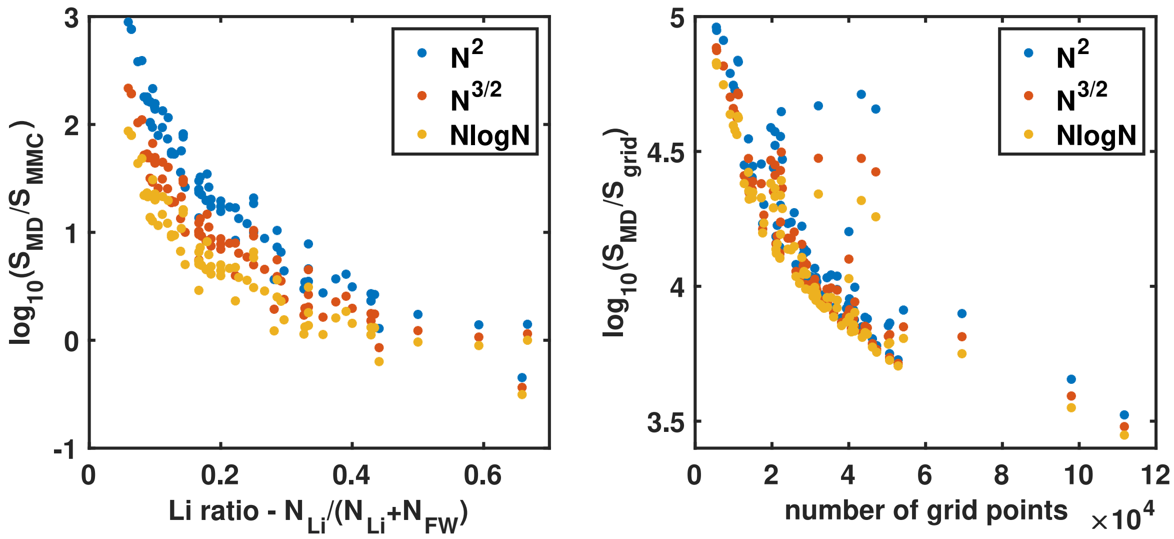

Figure S7: Speed-up dependencies presented for different scaling orders. Left: Log-scale speed-up ratios of MMC compared to MD depending on the Li-ratio. Right: Log-scale speed-up ratios of the grid sampling routine compared to MD depending on the number of 0.2 Å grid points.

References

(S1) Perdew, J. P.; Burke, K.; Ernzerhof, M. Generalized Gradient Approximation Made Simple. *Phys. Rev. Lett.* **1996**, *77*, 3865–3868.

(S2) Campañá, C.; Mussard, B.; Woo, T. K. Electrostatic Potential Derived Atomic Charges for Periodic Systems Using a Modified Error Functional. *J. Chem. Theory Comput.* **2009**, *5*, 2866–2878.

(S3) Kühne, T. D.; Iannuzzi, M.; Del Ben, M.; Rybkin, V. V.; Seewald, P.; Stein, F.; Laino, T.; Khaliullin, R. Z.; Schütt, O.; Schiffmann, F.; Golze, D.; Wilhelm, J.; Chulkov, S.; Bani-Hashemian, M. H.; Weber, V.; Borštnik, U.; Taillefumier, M.; Jakobovits, A. S.; Lazzaro, A.; Pabst, H.; Müller, T.; Schade, R.; Guidon, M.; Andermatt, S.; Holmberg, N.; Schenter, G. K.; Hehn, A.; Bussy, A.; Belleflamme, F.; Tabacchi, G.; Glöß, A.; Lass, M.; Bethune, I.; Mundy, C. J.; Plessl, C.; Watkins, M.; VandeVondele, J.; Krack, M.; Hutter, J. CP2K: An electronic structure and molecular dynamics software package - Quickstep: Efficient and accurate electronic structure calculations. *J. Chem. Phys.* **2020**, *152*, 194103.

(S4) VandeVondele, J.; Hutter, J. Gaussian basis sets for accurate calculations on molecular systems in gas and condensed phases. *J. Chem. Phys.* **2007**, *127*, 114105.

(S5) Goedecker, S.; Teter, M.; Hutter, J. Separable dual-space Gaussian pseudopotentials. *Phys. Rev. B* **1996**, *54*, 1703–1710.

(S6) Pollock, E. L.; Glosli, J. Comments on P3M, FMM, and the Ewald method for large periodic Coulombic systems. *Comput. Phys. Commun.* **1996**, *95*, 93–110.
